# Hypothalamic–Pituitary–Adrenal Axis Dysfunction in People With Cancer: A Systematic Review

**DOI:** 10.1002/cam4.70366

**Published:** 2024-11-21

**Authors:** Natalie G. Kanter, Sarah Cohen‐Woods, David A. Balfour, Morton G. Burt, Alison L. Waterman, Bogda Koczwara

**Affiliations:** ^1^ College of Medicine and Public Health Flinders University Bedford Park South Australia Australia; ^2^ Flinders University Institute for Mental Health and Wellbeing Flinders University Bedford Park South Australia Australia; ^3^ College of Education, Psychology, and Social Work Flinders University Bedford Park South Australia Australia; ^4^ Flinders Centre for Innovation in Cancer College of Medicine and Public Health, Flinders University Bedford Park South Australia Australia; ^5^ Southern Adelaide Diabetes and Endocrine Services Flinders Medical Centre Bedford Park South Australia Australia; ^6^ Department of Medical Oncology Flinders Medical Centre Bedford Park South Australia Australia

**Keywords:** glucocorticoids, neoplasm, physiological, pituitary–adrenal system, stress, systematic review

## Abstract

**Purpose:**

Cancer can be a source of significant psychological and physical stress. Prolonged stressful stimuli can influence the stress response, mediated by the hypothalamic–pituitary–adrenal (HPA) axis. However, there is limited literature investigating HPA axis function in patients with cancer.

**Methods:**

A systematic literature review of case–control studies was conducted comparing individuals with and without cancer examining the HPA axis function. Databases (MEDLINE, PubMed, Scopus) were searched from inception to May 2023.

**Results:**

Seventeen studies met eligibility criteria: nine unstimulated‐cortisol studies and eight reporting the effect of HPA stimulation or suppression. Sixteen studies reported altered levels of HPA function in cancer patients relative to controls, including 13 reporting increased baseline or hyperactive cortisol responses, and four—decreased baseline cortisol or blunted cortisol responses, two of which had patient groups with now known cortisol‐suppressing treatments. HPA dysfunction was observed in patients of both sexes, diverse ages, stages of cancer and cancer treatments. Six papers reported on clinical outcomes with cases experiencing higher levels of fatigue, stress, poor memory, poor well‐being and disturbed sleep. There was significant heterogeneity in methodologies across the studies.

**Conclusion:**

HPA dysfunction was common in patients with cancer relative to cancer‐free controls. The majority of studies in cancer reported an increased baseline cortisol and increased response to HPA stimulation. There is a need for well‐powered studies using standardised methodology examining the mechanisms of HPA dysregulation and their health outcomes, to enable the development of appropriate tools for the diagnosis and management of HPA dysfunction in cancer.

## Abstract 2

This systematic review of 17 studies comparing HPA axis function in individuals with cancer as compared to controls suggests that HPA dysregulation is common in people with cancer. There was significant variability in methodologies used to measure HPA dysregulation, which limits the interpretation of findings, and few only studies examined the impact of HPA dysregulation on patient outcomes. There is a need for studies that use a standardised approach to HPA axis measurement and examine patient outcomes to determine its clinical impact.

## 1 Introduction

Cancer diagnosis and its subsequent treatments can act as a significant physical and psychological stressors [1]. The resulting heightened stress can occur over a prolonged period and can be associated with complex changes in cardiometabolic and endocrine function. This includes dysregulation of the hypothalamic–pituitary–adrenal (HPA) axis, a central component of the stress response system [2].

The typical stress response to endogenous or exogenous stressors involves physiological and behavioural changes designed to first facilitate a ‘flight, fight or freeze’ response and then accommodate a return to a homeostatic baseline, through an intricate interplay between the HPA axis, the autonomic and central nervous system centres and their target tissues and organs [3]. The initial response to a stressful stimulus is mediated by the activation of the sympathetic nervous system, with the release of catecholamines that increase heart and respiration rate, ready to face ‘danger’. This is followed by the release of corticotropin‐releasing hormone (CRH) from the hypothalamus, triggering the release of adrenocorticotropic hormone (ACTH) and cortisol production to maintain the response until the threat has passed. These hormonal changes stimulate the release of glucose [4]. Increased levels of cortisol suppress further CRH and ACTH release [3, 5]. This feedback loop allows for homeostasis to be achieved again and ends the stress response and ongoing HPA activation. Chronic stress states can lead to either over or under‐expression of cortisol, and suppression or overstimulation of the HPA axis [6]. In either situation, the HPA axis is not able to react optimally to a stimulus. This dysregulation of the HPA axis has been reported in various conditions including depression, anxiety, posttraumatic stress disorder, obesity and other metabolic syndrome profiles and cancer [7, 8, 9, 10].

We identified over 70 systematic reviews published in the past 5 years on HPA axis dysregulation across diverse conditions, including depression, anxiety, post‐traumatic stress disorder, autism spectrum disorder, Parkinson's disease and endometriosis‐associated pain [10, 11, 12, 13, 14]. There were no systematic reviews, however, that focussed on the function of the HPA axis in cancer.

To address this gap, we examined the evidence on HPA axis function in people with cancer as compared to cancer‐free controls. Specifically, the objectives of the review were to examine (1) the prevalence and nature of HPA axis dysregulation in cancer and (2) the impact of HPA axis dysregulation on healthcare outcomes.

## 2 Methods

### 2.1 Search Strategy

A systematic review was performed according to the preferred reporting items for systematic reviews and meta‐analyses (PRISMA) guidelines. With the support of a librarian, an electronic literature search of MEDLINE, PubMed and Scopus, from inception until 19 May 2023 was conducted. Medical subheadings (MeSH) and keywords were searched. The search terms used were different variations of ‘Hypothalamic pituitary adrenal axis’ OR ‘HPA axis’ OR ‘Cortisol’ AND ‘cancer’ (See Appendix S1 for the full search strategy). These search terms were combined using Boolean operators OR between similar terms and AND between the two main search concepts, HPA axis and cancer.

### 2.2 Selection Criteria

Inclusion criteria were as follows: people over 18 years of age; written in English; a patient group with a diagnosis of cancer (any‐stage current diagnosis, or cancer survivor) and a control group of either healthy volunteers or patients with nonmalignant disease; measurement of HPA axis activity (e.g., salivary, blood or urine cortisol measurements). Laboratory methodologies, study protocols, case studies, commentaries, editorials, reviews and opinion pieces were excluded.

### 2.3 Data Extraction, Quality Appraisal and Data Analysis

Two independent reviewers evaluated each abstract, then each full text that passed the abstract screening stage, following the PRISMA guidelines. Where there were discrepancies, they were discussed between the two independent reviewers and escalated to the whole research team where required. A quality appraisal of the studies was conducted by two independent reviewers using the JBI critical appraisal checklist for case–control studies [15].

For studies that met the full inclusion criteria, the following data were extracted: author, year, title, study objective, number of participants, definitions of case and control, cortisol measurement method and intervention type (if relevant). For a summary of key results see Tables 1 and 2.

**TABLE 1:** The impact on the HPA axis in a cancer population in unstimulated cortisol case–control studies.

Author, year, title	Study objective	Population (cancer type, cancer treatment, other relevant)	Cortisol measurement method	Study result
Arlt et al. [17] (1997) Germany Frequent and frequently overlooked: treatment‐induced endocrine dysfunction in adult long‐term survivors of primary brain tumours	To assess frequency and clinical impact of endocrine dysfunction in adult long‐term survivors of primary brain tumours outside the hypothalamic–pituitary region	Total: 62 31 long‐term survivors of primary malignant brain tumours (not affecting hypothalamic–pituitary region; previously treated tumour) versus 31 controls with peripheral nervous system disease Treatment included combinations of surgery, chemo and radiotherapy Matched by age and gender 44.1 ± 11.5 years old versus 44.1 ± 11.1 years old	Serum samples measured between 8 am and 10 am	‐ Cases had over 25% lower mean serum baseline concentration than controls (*p* < 0.01) ‐ 77% of cases were found to have at least one neuroendocrine abnormality at baseline compared to only 6% of patients ‐ Neuroendocrine dysfunction symptoms (e.g., difficulties concentrating, poor memory, weight gain, fatigue, poor libido) were more frequent in cases versus controls, as per the patient questionnaire. Various symptoms were reported up to six times more frequently in cases than controls
Bernabe et al. [18] (2012) Brazil Increased plasma and salivary cortisol levels in patients with oral cancer and their association with clinical stage	To evaluate plasma and salivary cortisol in patients with oral and oropharyngeal squamous cell carcinoma (SCC), oral leucoplakia, smokers and/or drinkers and healthy control subjects and correlate data with clinicopathological parameters	Total: 120 34 patients with active oral SCC, 17 patients with active oropharyngeal SCC, 17 patients with oral leucoplakia (precancerous), 27 volunteers with smoking/alcohol abuse (patient with risk of cancer) and 25 healthy volunteers Mean age 60.25 years old, gender matched	Serum and salivary samples measured between 8 am and 10 am	‐ Patient with oral SCC had the highest mean plasma cortisol levels, and more than 20% greater than healthy controls (*p* = 0.04) ‐ Salivary cortisol levels followed a similar pattern to plasma cortisol, patients with oral SCC having the highest levels—nearly 50% greater than healthy controls (*p* = 0.005) ‐ More advanced oral SCC was associated with higher cortisol levels than early‐stage oral SCC (*p* = 0.015)
Engin et al. [19] (2016) Turkey Circulating IL‐6 and neopterin concentrations link cell‐mediated immunity and tumour stage in patients with gastro‐intestinal adenocarcinoma: relevance to the pituitary–adrenal axis and pituitary–thyroid axis	To identify the interactions between serum neopterin, cortisol, IL‐6, IL‐10 and TSH levels in the progression of gastrointestinal carcinoma	Total: 109 67 patients with gastrointestinal cancer versus 42 cancer‐free patients with chronic cholecystitis controls 61.26 ± 1.52 years old versus 55.52 ± 1.97 years old Both groups underwent gastrointestinal surgery (with curative intent)	Serum samples measured in the morning	‐ Mean serum cortisol levels in cases were over 30% higher than the mean cortisol level in controls (*p* = 0.001) ‐ More advanced disease was associated with higher cortisol levels ‐ Data indicated that cortisol, IL‐6 and neopterin values of cases were influenced by tumour presence and progression
Mazzoccoli et al. [24] (2011) Italy Determination of whole‐body circadian phase in lung cancer patients: Melatonin versus cortisol	To evaluate a feasible methodology for the determination of whole‐body circadian phase in lung cancer for planning of chronotherapy (appropriate timing of treatment)	Total: 20 Nine patients with metastasised non‐small cell lung cancer (NSCLC) versus 11 controls (with back pain or irritable bowel syndrome) Age 43–63 years old versus 35–53 years old; BMI matched 20–30; Gender (M)	Serum levels measured every 4 h for 24 h	‐ Cortisol serum levels measured in cases were 2× higher than those in controls (*p* < 0.001) ‐ Near‐awakening levels of cortisol were the highest in the 24 h period for both cases and controls, as expected ‐ Cortisol serum levels followed a similar 24 h pattern in both cases and controls with no statistically significant difference in pattern
Mazzoccoli et al. [25] (2011) Italy Chronodisruption in lung cancer and possible therapeutic approaches	To evaluate time‐related variations of neuroendocrine and immune system components (GH‐IGF1 axis, hypothalamus–pituitary–thyroid axis, melatonin, cortisol, lymphocyte subsets and IL2) in lung cancer patients compared to healthy controls	Total: 20 Nine patients with metastasised non‐small cell lung cancer NSCLC versus 11 healthy controls Age 43–63 years old versus 35–53 years old; BMI matched 20–30; Gender (M)	Serum levels measured every 4 h for 24 h	‐ Mean serum cortisol levels in cases were nearly 1.5× greater than in controls (*p* < 0.001) ‐ Controls showed greater variation in circadian cortisol rhythm, whereas cases showed loss of normal circadian variation of cortisol levels, with a blunted cortisol pattern (*p* < 0.01)
Mazzoccoli et al. [26] (2010) Italy Altered time structure of neuro‐endocrine‐immune system function in lung cancer patients	To evaluate the difference between case versus control in 24 h secretory profile of neuro‐immune‐endocrine system	Total: 20 10 patients with untreated NSCLC versus 10 healthy controls Age matched 45–65 years old and gender (M) and BMI (25–30)	Serum levels measured every 4 h for 24 h	‐ Mean cortisol levels were nearly 50% greater in cases than in controls (*p* < 0.05) ‐ In controls, cortisol presented following normal circadian rhythmicity, however, in cases, cortisol was shown to have lost its circadian rhythmicity, showing a blunted cortisol pattern
Oh et al. [27] (2019) Korea Altered hypothalamus–pituitary–adrenal axis Function: A Potential Underlying Biological Pathway for Multiple Concurrent Symptoms in Patients With Advanced Lung Cancer	To examine whether HPA axis dysfunction underlies concurrent multiple symptoms in patients with advanced cancer	Total: 93 46 patients with active lung cancer versus 47 healthy controls Age: 64.3 ± 9.2 years old versus 62 ± 4.6 years old Symptoms measured using MDASI	Salivary samples measured after waking and night‐time	‐ Cases showed reduced cortisol awakening response (CAR) and flatter diurnal slopes (*p* < 0.001) versus controls, hence a blunted cortisol pattern ‐ Cases with metastases, (i.e., more severe disease) had lower CAR than cases with primary disease only (*p* = 0.03) ‐ Each MDASI symptom varied widely in degree of association with HPA axis function ‐ Symptoms of fatigue, pain, disturbed sleep, SOB and anorexia were associated with altered HPA axis function
Porter et al. [28] (2003) USA Cortisol Levels and Responses to Mammography Screening in Breast Cancer Survivors: A Pilot Study	To compare baseline cortisol levels (salivary, diurnal slope and reactivity) in response to a mammogram in breast cancer survivors versus women without a cancer history	Total: 54 33 breast cancer survivors (3–5 years. post treatment) versus 21 controls (scheduled for routine follow‐up mammogram) Age 56.3 ± 10.8 years old versus 54.4 ± 10 years old, gender (F)	Salivary samples taken for 3 consecutive days 1 month before the mammogram, then day before, day of and day after the mammogram Salivary samples taken 6 times daily	‐ Cases had nearly 50% higher cortisol levels at baseline than controls (*p* < 0.001) ‐ Cortisol levels followed an increasing pattern as time approached day of mammogram ‐ Obvious variation with cortisol levels and flat or steep diurnal patterns associated with various treatments of the breast cancer undertaken ‐ There was no significant difference in group baseline stress (*p* > 0.67), however the difference in stress levels day of mammogram—cases had higher stress levels than controls (*p* = 0.14)
Weinrib et al. [32] (2010) USA Diurnal cortisol dysregulation, functional disability and depression in women with ovarian cancer	To investigate alterations in diurnal cortisol rhythm in ovarian cancer patients, and potential links with depression, life stress and functional disability	Total: 210 100 patients with ovarian cancer undergone surgical treatment versus 77 patients with benign disease versus 33 healthy controls	Salivary samples measured upon wakening, late afternoon and before bedtime for 3 days before surgery	‐ Cases and controls did not differ in their morning cortisol levels ‐ Cases had higher cortisol in the afternoon than healthy controls, but were not significantly different from controls with benign disease (*p* < 0.001 and *p* = 0.07) ‐ Cases had significantly higher nocturnal cortisol levels than controls with benign disease and with healthy controls (*p* = 0.022 and *p* < 0.0001) ‐ Diurnal cortisol pattern in cases was lower than in controls with benign disease and lower than healthy controls, showing a blunted cortisol pattern (*p* = 0.023 and *p* < 0.0001) ‐ High nocturnal cortisol and flatter diurnal pattern that were present in the cases were associated with greater fatigue, poorer performance and poorer physical well‐being (*p* < 0.05) ‐ There was no significant association between these variables in women with benign disease

Abbreviations: ACTH = adrenocorticotropic hormone; CAR = cortisol awakening response; CRH = cortisol‐releasing hormone; HPA = hypothalamic–pituitary–adrenal.

**TABLE 2:** The impact on the HPA axis in a cancer population in stimulated cortisol case–control studies.

Author, year, title	Study objective	Population (cancer type, cancer treatment, other relevant)	Cortisol measurement method	Intervention	Study result
Andreano et al. [16] (2012) USA Effects of breast cancer treatment on the hormonal and cognitive consequences of acute stress	To test if disruption (via cortisol‐activating stressor ‘cold pressor stress’) of both ovarian and glucocorticoid systems associated with breast cancer may interact in their effect on cognition (working memory, verbal paired associated memory and narrative recall)	Total: 40 20 patients with breast cancer treated with Lupron (nil recurrent or with stable tumour) versus 20 naturally cycling controls Matched by age (27–49/26–48 years old) and gender (F) 50% of each group underwent CPS; other 50% did not (immersed hand in warm water for 3 min instead)	Salivary (1× in the first 10 min, +10, +20, +30 min post CPS; 1 × 1 week later)	‘Cold Pressor Stress’ (immersing hand in ice water for 3 min to activate cortisol sympathetic response)	‐ Controls had a significant increase in cortisol and difference between cold and warm water stimuli (*p* < 0.05) versus cases had no significant increase or difference in cortisol levels between cold and warm water stimuli (*p* < 0.05) ‐ Hence showing a blunted cortisol response in case versus control ‐ No significant difference in verbal paired association or working memory reported ‐ In the relatively emotionally arousing story recall, there was delayed recall in cases compared to controls
Lissoni et al. [20] (2005) Italy Cortisol response to an acute injection of Il‐2 in healthy subjects and cancer patients: A first immunoneuroendocrine standardised clinical test to explore the interactions between immune and neuroendocrine systems	To evaluate cortisol response to an acute IL‐2 injection in healthy subjects and metastatic cancer patients, to standardise a clinical neuro‐endocrino‐immune test capable of documenting possible alterations of the link between neuroendocrine and immune systems	Total: 20 10 patients with current metastatic disease versus 10 healthy controls Age and gender matched (cases 48–65 years old) Cancer types included renal cell carcinoma, colorectal cancer, hepatocarcinoma, malignant melanoma	Blood levels at 0, +2, +4, +6 h post IL‐2 injection	Il‐2 injection Controls (baseline and low dose 3 million IU) Cases (low dose 3 million IU and high dose 9 million IU)	‐ Controls mounted expected response, increase cortisol levels post Il‐2 injection from baseline ‐ Cases did not show increase in cortisol at low dose Il‐2, difference between cortisol response of cases versus controls at low dose Il‐2 was statistically significant (*p* < 0.001) ‐ Cases (8 out of 10) showed increase in cortisol response at high dose Il‐2, however, mean cortisol levels remained lower than in cases at low dose Il‐2 ‐ Cases had blunted cortisol response to Il‐2
Lizcano et al. [21] (2011) Spain Thyroid hormone therapy modulates hypothalamo–pituitary–adrenal axis	To observe the influence of thyroid hormone therapy on the HPA axis To measure changes in plasma ACTH and cortisol levels in response to human corticotropin‐releasing‐hormone (hCRH)	Total: 21 14 athyreotic women due to thyroid cancer treatment studied before and after thyroid suppression therapy with thyroxine (T4) versus 7 healthy women as controls Age 42.9 ± 3.2 years old versus 36.2 ± 8.2 years old in control group	Serum samples at −45, −30, −15 min and + 15, +30, +45, +60, +75 min pre and post hCRH administration Test conducted before T4 therapy and after 2 months in cases	hCRH (100 microg)	‐ Ablation and suppression therapy with T4 is a thyroid cancer treatment ‐ No significant difference in baseline ACTH or cortisol levels observed in cases compared to controls pre‐T4 therapy ‐ Post CRH, ACTH and cortisol response were significantly greater in cases compared to controls (*p* < 0.05) ‐ After T4 therapy, cases had an increase in ACTH baseline levels but similar cortisol levels at baseline than before T4 therapy ‐ Post CRH in post‐T4 therapy cases, there was a significant increase in ACTH and cortisol (p < 0.05) (i.e., a hypersensitive response) ‐ Cases always mounted a greater response, evident by increased ACTH and cortisol levels post hCRH injection, when compared to controls
Lundstrom et al. [22] (2000) Sweden Aspects of delayed chemotherapy‐induced nausea Dexamethasone and adrenal response patterns in patients and healthy volunteers	To investigate whether the recovery of the HPA axis differs between patients with gynaecological cancer and healthy female volunteers	Total: 15 Five patients with gynaecological cancer (being treated with chemotherapy receiving 8 or 20 mg of dexamethasone during two consecutive chemotherapy cycles) versus 10 healthy F volunteers (receiving single dose of 8 mg of dexamethasone) Age matched—mean 57 years old versus 53 years old	Urinary‐free cortisol (UFC) Collected 24 h before, and 4 days (controls)/ 5 days (cases) after dexamethasone infusion	Dexamethasone IV (8 mg or 20 mg)	‐ In both cases and controls, injections of dexamethasone led to a decline in endogenous cortisol levels in 24 h and a subsequent recovery in the next 24 h period (*p* < 0.05) ‐ Therefore, the pattern of recovery in case versus control is similar with no statistically significant difference
Lutz et al. [23] (2000) Germany Adrenocortical function in patients with macro‐metastases of the adrenal gland	To evaluate the adrenocortical responsiveness to exogenous adrenocorticotropin (ACTH) in a series of patients and controls	Total: 35 28 patients with bronchogenic carcinoma (11 without adrenal metastases, 8 with unilateral adrenal metastases, 9 with bilateral adrenal metastases) versus 7 healthy controls Age and gender matched (55–70 years old vs. 31–39 years old) Subgroup testing—others could not be studied due to confounding factors 12 patients with bronchogenic carcinoma (6 without metastases, 3 with unilateral and 3 with bilateral)	ACTH Serum cortisol at time − 30, 0, +30, +60, +90 min Dexamethasone Serum cortisol at 0800 h (after administration of dexamethasone at 2300 h the night before)	ACTH (250 microg IV) Dexamethasone suppression test (3 mg oral)	‐ Patients had symptoms suggestive of adrenal insufficiency like weakness, fatigue, anorexia, nausea/vomiting ‐ However, cases had higher baseline and post ACTH stimulated cortisol levels than controls ‐ Cortisol increases were more pronounced the greater the metastatic involvement was ‐ Dexamethasone suppresses the HPA axis which was evident in controls and cases. However, ACTH was suppressed more in controls, resulting in cases having higher‐post‐dexamethasone cortisol levels, a sign of HPA dysfunction
Reimondo et al. [29] (2017) Italy Effects of mitotane on the hypothalamic–pituitary–adrenal axis in patients with adrenocortical carcinoma	To assess the HPA axis in patients with adrenocortical cancer (ACC) receiving mitotane (a drug used to treat ACC, potentially causing adrenal insufficiency)	Total: 26 16 patients with resected ACC versus 10 controls with primary adrenal insufficiency Age and gender matched (cases 35–70 years old vs. 32–63 years old) Patients on adjuvant treatment with mitotane (steroidogenesis inhibitor and antineoplastic medication) after radical surgical resection of ACC	Blood samples of ACTH and cortisol were taken at −15, 0, +15, +30, +45, +60 min post‐CRH injection	hCRH (100microg IV)	‐ In cases, baseline cortisol was reduced versus controls ‐ After CRH, median value of percent ACTH increment was 85% and median value of percent cortisol increment was 0% ‐ ACTH levels were higher in controls versus cases at baseline (*p* < 0.05), ‐ Both cases and controls followed normal pattern post CRH, with cases having lower values but also had lower starting points at baseline
Seppelt et al. [30] (1998) Germany Cortisol, immune status and patients' coping in primary breast cancer	To determine if there are any differences in baseline and after stimulation with CRH cortisol levels between different tumour groups	Total: 32 10 patients with breast carcinoma, 10 patients with benign breast tumours and 12 healthy controls (no tumours) Mean ages 56 versus 47 versus 32 years old Measured coping ability of patients via Mental Adjustment to Cancer Scale	Salivary cortisol pre‐CRH and post‐CRH at −15, −15, +15, +30, +45, +60, +75, +90, +105 min	hCRH (IV)	‐ Malignant cases showed significantly high concentration differences in cortisol levels pre and post hCRH and increase in baseline cortisol concentration ‐ Higher cortisol level in malignant cases was associated with the greatest percentage of weak fighting spirit, helplessness, hopelessness, strong anxiety and disease avoidance behaviours reported
Van Waas et al. [31] (2012) The Netherlands Adrenal function in adult long‐term survivors of nephroblastoma and neuroblastoma	To assess adrenal function and its metabolic effects (effect of cortisol on lipids and insulin resistance) in survivors after adrenalectomy	Total: 152 67 survivors of nephroblastoma, 36 survivors of neuroblastoma versus 49 controls Age and gender matched Survivors of childhood cancer, > 5 years after cessation of treatment	Serum cortisol samples were taken at 0, +30, +60 min after Synacthen	Synacthen solution ACTH (1 microg IV)	‐Adrenal insufficiency was not present in survivors ‐ Baseline cortisol and after Synacthen test were higher in survivors with unilateral adrenalectomy than those with both adrenals intact (*p* = 0.002)

Abbreviations: ACTH = adrenocorticotropic hormone; CAR = cortisol awakening response; CRH = corticotropin‐releasing hormone; HPA = hypothalamic–pituitary–adrenal.

## 3 Results

**FIGURE 1:**
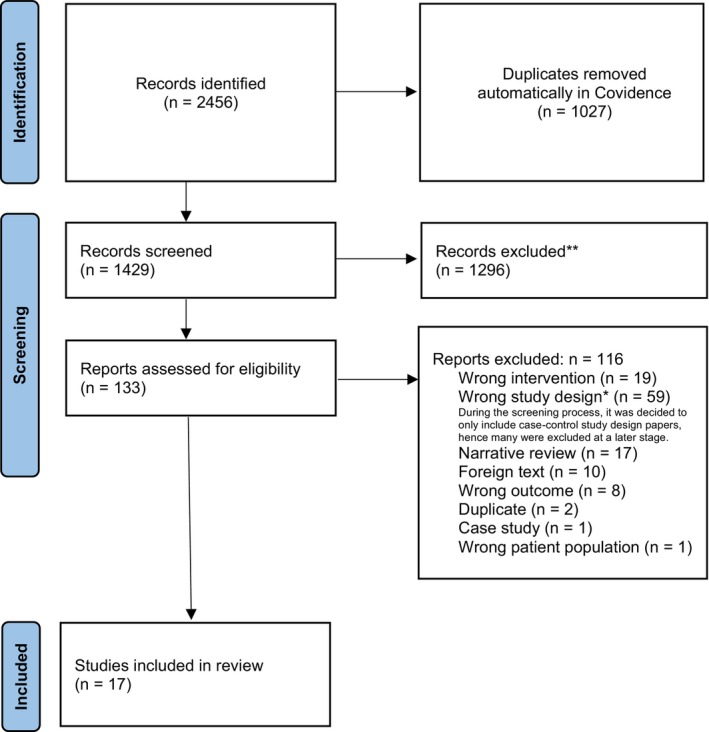
PRISMA flow diagram representing the stages of the literature search.

The initial search resulted in 2456 papers, with 1027 duplicates identified and removed. The remaining 1429 papers had their abstracts screened by two independent reviewers, resulting in 133 papers selected for full‐text review. Of those, 17 papers were included in the review (see Figure 1). The 17 included papers were published between 1997 and 2019 across nine countries including the United States (three), Germany (three), Italy (five), and one each from The Netherlands, Sweden, Spain, Brazil, Turkey and Korea [16, 17, 18, 19, 20, 21, 22, 23, 24, 25, 26, 27, 28, 29, 30, 31, 32].

All papers were case–control studies, comparing patients with cancer (14) [18, 19, 20, 21, 22, 23, 24, 25, 26, 27, 29, 30, 32] or cancer survivors (three) [17, 28, 31] to controls either patients with nonmalignant disease (four studies), [17, 19, 24, 29] healthy controls (10 studies) [16, 20, 21, 22, 23, 25, 26, 27, 28, 31] or both (three studies) [18, 30, 32]. In 11 of the 17 papers, the subjects were matched by age and gender; the other papers did not state any matching [16, 17, 19, 20, 21, 22, 23, 24, 25, 26, 27]. The studies included a variety of cancers, including lung (five), breast (three), adrenal (two), gynaecological (two) and single papers on brain, oral and oropharyngeal, gastrointestinal, thyroid cancer and patients with metastatic disease from various cancers.

There were nine observational studies [17, 18, 19, 24, 25, 26, 27, 28, 32] and eight studies that assessed the dynamic cortisol response to a physiologic (cold‐press stressor where the participants hands were immersed in icy‐cold water) [16] or pharmacologic stimulator (CRH; [21, 29, 30] adrenocorticotropin (ACTH); [23, 31] interleukin‐2 [20]) or suppressor (dexamethasone [22]) on HPA activity.

Sample sizes varied from 15 to 210, with the median number of participants being 40 and a total of 1084 participants across the 17 papers. Ages ranged from 18 to 70 years, with a total of 563 female participants and 367 male participants reported, however, one paper did not outline gender distribution in participants [19], and another only explicitly stated gender distribution for the cases and that controls were gender‐matched [20]. The ethnic background or country of origin of the participants was not specified in any of the papers.

All studies used cortisol levels as a measure of HPA axis activity. Cortisol levels were taken from salivary samples (five papers), serum samples (nine papers), urine samples (one paper), or both salivary and serum samples (two papers). Cortisol measuring methods were very varied as well, especially in the time of day. Measurement protocols varied amongst the papers and included unstimulated morning samples, or interval cortisol measurements throughout a 24‐h period in the observational studies. The interventional studies measured cortisol levels as a response to the stimulation or suppression of the HPA axis, with great variability, including from 10 min to 5 days after the intervention, measuring in 10‐, 15‐, 30‐min, 2‐ or 24‐h intervals.

Quality appraisal was conducted separately for interventional and observational papers. The median score for the interventional papers was 8.5, with scores ranging from 6 to 9.5 out of 10. The most prominent reasons for lower quality scores were inconclusive evidence for the identification and rectification of confounding factors, and the lack of definitive identification criteria for cases and controls. The median score for the observational papers was 7.5, with scores ranging from 7 to 8 out of 8. In the lower‐scoring papers, the appropriate identification and rectification of confounding factors was often unclear.

### 3.1 Unstimulated Cortisol Studies

Nine papers reported only on single unstimulated cortisol levels and patterns of cortisol activity in various cancer populations relative to their control groups which included healthy volunteers or patients with benign disease. All studies reported abnormalities in HPA axis function in cases, with eight of the nine papers reporting a higher mean cortisol level in cases compared to controls [18, 19, 24, 25, 26, 27, 28, 32]. The difference in mean cortisol levels ranged from 20% to 200% greater levels in cases than in controls. The exception to this pattern was a study of 31 long‐term survivors of brain tumours, which had previously been treated with central nervous system (CNS) radiotherapy. The authors reported that the survivors had a lower mean morning cortisol level compared to the 31 controls [17]. They also reported that cases were up to six times more likely to experience symptoms of fatigue, weight gain, poor memory and concentration compared to the control group [17], (Table **1**).

Two of the nine studies compared the HPA axis and cortisol activity in populations with different stages of a disease. One study compared healthy controls, patients with cancer risk factors, patients with precancerous changes, and patients with active oral squamous cell carcinoma, while the other looked at patients with benign gastrointestinal disease and then different stages of gastrointestinal cancer [18, 19]. Both papers reported higher cortisol levels in cases compared to controls, and within the cases reported higher cortisol levels in more advanced diseased states and advanced cancer stages [18, 19].

Five studies measured cortisol at multiple time points. Of these, three studies of patients with active lung cancer compared to healthy controls [25, 26, 27], and one study of patients with surgically treated ovarian cancer compared to controls with either benign disease or healthy [32], reported reduced variation in the circadian rhythmicity of cortisol, meaning continued elevation of cortisol without the natural dip, (i.e., flatter diurnal slopes). This blunted type of HPA dysregulation was associated with fatigue, pain, disturbed sleep, poor performance and poor physical well‐being in the cases [27, 32]. The fifth study, also in patients with lung cancer did not show a significant difference in the circadian pattern of cortisol amongst its nine cases or the 11 controls with irritable bowel syndrome, but did report higher morning cortisol levels in the cases when compared to controls [24].

One study in breast cancer survivors and healthy controls, both scheduled for a routine mammogram, recorded the patients' subjective mood and stress through a questionnaire that asked them to pick one of nine words describing positive or negative affect and rank their level of stress from 0 to 100. The questionnaire was conducted along with cortisol measurements six times daily for three consecutive days 1 month before the mammogram, then the day before, the day of and the day after the mammogram. The authors reported no significant difference in group baseline mood or stress measurement, however—as the day before and the day of the mammogram approached—cases had higher cortisol levels and reported subjectively higher levels of stress compared to controls [28].

### 3.2 Stimulated Cortisol Studies

Eight papers reported the change in cortisol concentration in response to a physiologic or pharmacologic stimulator or inhibitor of the HPA axis (Table **2**).

Five manuscripts have reported the change in cortisol after parenteral CRH or ACTH.

Three studies reported a higher baseline mean cortisol level in cases, and an elevated cortisol response to CRH or ACTH, compared to the control group [21, 23, 30]. The elevated response was seen in patients before and during treatment for thyroid cancer, patients with active lung cancer with unilateral or bilateral adrenal metastases, and patients with active breast cancer [21, 23, 30]. The latter group included 10 patients with surgically proven malignant breast tumours, compared to 22 controls (10 patients with benign breast tumours and 12 patients with no tumours). The patients with malignant tumours reported feelings of weak fighting spirit, helplessness, hopelessness and anxiety compared to the controls [30].

A study including adult survivors of both childhood nephro‐ and neuro‐blastomas, 67 and 36 patients, respectively, did not show a difference in response to ACTH compared to their group of 49 healthy controls [31]. While not showing a difference in response to a stimulus, the cases did exhibit higher baseline cortisol levels, which were associated with insulin resistance and increased lipid concentrations [31].

Another study primarily investigating the effect of mitotane on pituitary function studied 16 patients prescribed mitotane as adjuvant therapy treatment for radically resected adrenocortical cancer compared to 10 controls with primary adrenal insufficiency [29]. They reported the cortisol increment after CRH was lower in cases as compared to controls [29].

Two studies measuring serum and salivary cortisol reported a blunted HPA response to a stressor [16, 20]. The first, which was a study of 20 patients with breast cancer on hormonal therapy, and 20 healthy controls, used a cold‐pressor stressor, by immersing the participant's hand into ice cold water, on half of all participants. In addition to a blunter HPA response, this study reported significantly impaired memory/retention of information in the cancer patient group [16]. The second paper demonstrating a blunted HPA response to a cytokine injection acting as the pharmacologic stressor included 10 healthy controls and 10 patients with extensive metastatic disease due to liver, kidney, colorectal or skin cancers [20].

Two papers reported on the degree of cortisol suppression after dexamethasone administration, a known suppressor and the HPA axis. One study reported an attenuated fall in plasma cortisol after dexamethasone in 28 patients with cancer with and without adrenal metastases. The cases maintained a higher cortisol level than the controls, as suppression in the population with cancer was not as great as in the seven noncancer controls [23]. A study of five patients with gynaecological cancers and 10 healthy controls reported an expected pattern of fall and subsequent rise of urinary cortisol levels after dexamethasone injection, with no significant difference in cortisol levels or variation between cases and controls [22].

## 4 Discussion

This systematic review of HPA axis function in people with cancer revealed emerging evidence of HPA dysregulation, including an association with worse health outcomes including fatigue, disturbed sleep, subjective stress and poorer performance. This dysregulation was evident in 16 out of 17 papers reviewed, with one small study in gynaecological cancers with just five cases and 10 controls being the exception, having not reported any difference between cases or controls [22]. HPA dysregulation was present across a range of cancer types, cancer stages, ages and genders of patients and cortisol measurement methods. The most common types of HPA dysfunction were increased baseline cortisol levels and increased HPA axis response to a stimulus. This was evident in eight of nine papers reporting on unstimulated cortisol levels and in five of eight papers reporting on stimulated cortisol responses [18, 19, 21, 23, 24, 25, 26, 27, 28, 30, 31, 32]. The papers reporting otherwise included four reporting the opposite effect, of lower cortisol in cases compared to controls. One included patients that had undergone previous CNS irradiation [17], however, radiotherapy to the CNS results in a significant radiation dose to the pituitary gland and is now a well‐recognised cause of hypopituitarism causing consequent cortisol deficiency [33]. Two studies used non‐standard tests of the HPA axis (a cold‐pressor stress test and IL‐2 injection); [16, 20] and one study investigated patients on mitotane, a now known cortisol metaboliser [29]. In the study, all patients were hypoadrenal and taking cortisone acetate replacement, and the dose of mitotane prescribed in patients was approximately double the control group. Knowing that mitotane induces the metabolism of cortisol, the biological significance of these differences in cortisol levels post CRH is uncertain [34].

Only six papers reported on patient outcomes in the context of HPA dysfunction showing an association between HPA dysregulation and poor memory, fatigue, disturbed sleep, anorexia, subjective stress and poorer performance [16, 17, 27, 28, 30, 32]. Given the emerging evidence for HPA dysregulation identified in this review, yet the relatively limited data on patient health outcomes, this is an area for further research. HPA dysfunction has been associated with various conditions that impact cancer survivors disproportionately, such as cardiovascular disease and diabetes, which could represent a mechanism for the observed increase in comorbid conditions [35, 36]. HPA dysfunction in a noncancer population has been associated with an increased rate of mortality and increased prevalence of chronic diseases such as hypertension, diabetes mellitus and cardiovascular disease [37]. Recent studies in other clinical scenarios have highlighted that even minor increases in cortisol secretion are associated with increased cardio‐metabolic morbidity and cardiovascular mortality [38, 39]. Further research on the impact of HPA axis dysregulation on cancer patient health outcomes, be that due to the cancer, cancer treatment, physiological or psychological stress or other factors, will be critical to understanding functional outcomes of such dysregulation.

Patients with cancer receiving therapies known to potentially cause HPA hypofunction such as cerebral or nasopharyngeal irradiation, immune checkpoint inhibitors or mitotane should be systematically screened with morning serum cortisol and adrenocorticotrophin (ACTH) measurements, alone or in combination with a short Synacthen test. Given the small sample size of studies in other clinical scenarios, as highlighted in this review, it is premature to recommend clinical testing at this time. However, the results of this systematic review indicate that HPA dysfunction may be prevalent, and this should be a topic of ongoing active research. Future studies of HPA dysregulation should include relevant patient health outcomes to adequately assess the clinical impact of this condition.

The studies examined were undertaken within different cancer types, where mechanisms behind HPA dysregulation may be diverse. Potential mechanisms include prolonged exogenous stress, chronic inflammatory processes, neuroendocrine disruption due to cancer itself and short‐ and long‐term effects of chemotherapy or other cancer treatments [40, 41, 42]. Many of these potential mechanisms coexist and their complex interplay has been referred to as allostatic load which has been postulated as a driver of many chronic conditions including cancer [43]. Future research should be designed to explore the underlying mechanisms behind HPA axis dysregulation in this patient population, to enable the identification of potential timepoints and methods of intervention, and/or prevention. By understanding how and when HPA axis dysfunction occurs in people with cancer, and understanding what kinds of outcomes it may predispose them to, it may help identify methods of measuring, treating or preventing such dysregulation. If, in turn, it is determined that cancer‐associated disruption of the HPA axis also influences symptom burden and health comorbidity outcomes, this knowledge could also improve broader health outcomes for patients.

It is important to highlight some limitations of this analysis. There was great methodological heterogeneity; there were diverse approaches to measuring HPA axis function, including a lack of measuring cortisol binding globulin in serum samples, measuring function in unstimulated, stimulated and suppressed states and timing of measurements where some papers only reported on single time point results which do not account for the substantial day‐to‐day variability in cortisol secretion. Case populations differed in cancer type and cancer stage, including those of survivorship status, but not all types and stages were represented. There was also variation in population sample size in the different papers. Despite some of these limitations, this review's unique strength is that all the included papers had a control group of healthy patients or patients with nonmalignant disease, improving our capacity to draw conclusions about the potential relationship between cancer and the function of the HPA axis.

To our knowledge, this is the first systematic review on HPA axis dysfunction in cancer. Future research should focus on conducting large observational and case–control studies, using standardised cortisol measurement and intervention methods to enable more direct comparisons between results. Ideally, future studies should also include a comprehensive assessment of clinical outcomes including patient‐reported outcomes to holistically assess the impact of HPA dysfunction on the individual.

## 5 Conclusion

Various forms of HPA axis dysregulation have been reported, with most studies reporting people with cancer have increased baseline cortisol levels and increased cortisol response to a stimulator of the HPA axis relative to their cancer‐free controls. Further research is needed to examine more definitively the type of HPA dysregulation observed in people with cancer, and negative health outcomes associated with such HPA axis dysregulation. This is critical to determine if it is necessary to screen for dysregulation, and/or if a timely intervention could change its course and improve health outcomes.

## Author Contributions

**Natalie G. Kanter:** conceptualization (equal), formal analysis (equal), methodology (equal), writing – original draft (equal), writing – review and editing (equal). **Sarah Cohen‐Woods:** conceptualization (equal), methodology (equal), writing – review and editing (equal). **David A. Balfour:** formal analysis (equal), writing – review and editing (equal). **Morton G. Burt:** formal analysis (equal), writing – review and editing (equal). **Alison L. Waterman:** formal analysis (equal), writing – review and editing (equal). **Bogda Koczwara:** conceptualization (equal), formal analysis (equal), methodology (equal), resources (equal), supervision (equal), writing – review and editing (equal).

## Conflicts of Interest

The authors declare no conflicts of interest.

## Precis

Hypothalamic–pituitary–adrenal (HPA) axis dysfunction is commonly observed in individuals with cancer as compared to controls. Future research using standardised methods of HPA axis assessment correlating with clinical outcomes is needed.

## Supporting information


Appendix S1.

## Data Availability

The datasets generated during and/or analysed during the current study are available from the corresponding author on reasonable request.
