# Correction to Hypothalamic–Pituitary–Adrenal Axis Dysfunction in People With Cancer: A Systematic Review

**DOI:** 10.1002/cam4.70504

**Published:** 2024-12-15

N. G. Kanter, S. Cohen‐Woods, D. A. Balfour, M. G. Burt, A. L. Waterman, B. Koczwara. Hypothalamic–Pituitary–Adrenal Axis Dysfunction in People With Cancer: A Systematic Review. *Cancer Medicine* 13, no. 22 (2024):e70366, https://doi.org/10.1002/cam4.70366.

1. In the authors list, the addition of a middle initial for David Balfour was incorrect. The name should read **David Balfour**.
2. In the results section of the abstract, the number of studies was incorrect and should be changed from 13 to 12. The correct sentence should read: **Sixteen studies reported altered levels of HPA function in cancer patients relative to controls, including 12 reporting increased baseline or hyperactive cortisol responses, and four—decreased baseline cortisol or blunted cortisol responses, two of which had patient groups with now known cortisol‐suppressing treatments**.
3. In the first sentence of the introduction, the presence of “a” is incorrect. The sentence should read: **Cancer diagnosis and its subsequent treatments can act as significant physical and psychological stressors**.

We apologize for these errors.

