# Supplementary material for: Hypothalamic–Pituitary–Adrenal Axis Dysfunction in People With Cancer: A Systematic Review
Source: Cancer Med. 2024 Nov 21;13(22):e70366. doi: 10.1002/cam4.70366 (PMC11579619; doi:10.1002/cam4.70366)
Supplement: Supplementary file 1 — Appendix S1. [file CAM4-13-e70366-s001.docx]

## Appendix 1 – Search Strategy

1. Pituitary-Adrenal System/
2. “HPA Axis” or “hypothalam* pituitary adrenal” or “hypothalamic-pituitary-adrenal axis”
3. Cortisol or “cortisol level*” or “cortisol pattern*” or “cortisol awakening response*” or “morning cortisol level*” or “diurnal cortisol level*” or “diurnal cortisol pattern*” or “diurnal cortisol rhythm*”
4. Neoplasms/
5. Cancer or malignan* or tumo*
6. 1 or 2 or 3
7. 4 or 5
8. 6 and 7
